# Importance of duodenal bulb biopsies in children for diagnosis of celiac disease in clinical practice

**DOI:** 10.1186/1471-230X-9-78

**Published:** 2009-10-16

**Authors:** Mohsin Rashid, Andrea MacDonald

**Affiliations:** 1 Division of Gastroenterology and Nutrition, Department of Paediatrics, Faculty of Medicine, Dalhousie University, IWK Health Center, Halifax, Nova Scotia, Canada

## Abstract

**Background:**

The patchy nature of villous lesion in celiac disease is increasingly being recognized. Current guidelines recommend four endoscopic duodenal mucosal biopsies from the second or more distal part of the duodenum to confirm the diagnosis of celiac disease. The purpose of the study was to investigate the usefulness of duodenal bulb mucosal biopsies in confirming the diagnosis of celiac disease in everyday clinical practice.

**Methods:**

All patients with a positive tissue-transglutaminase antibody requiring biopsy-confirmation of celiac disease over a two-year period were studied. Two endoscopic biopsies were taken from the duodenal bulb and four biopsies from the second (or distal) part of the duodenum.

**Results:**

Thirty-five patients were included, mean age 8.1 (± 4.7) years. Thirty-one (88.6%) patients had abnormal distal duodenal biopsies, one had Marsh type 1, one had Marsh type 2 and twenty-nine had Marsh type 3 lesion. All but two patients with abnormal distal duodenal biopsies also had abnormal bulb biopsies. Four (11.4%) patients had normal distal duodenal biopsies but abnormal bulb biopsies. Of these, one patient had Marsh type 2 and three had Marsh type 3 lesion. The distal duodenum was also grossly normal in these four patients. The histological diagnosis of celiac disease would not have been possible in these four cases with distal duodenal biopsies only.

**Conclusion:**

The lesion in celiac disease in children can be patchy with duodenal bulb mucosa being the only area showing histological changes. The recommendations regarding the site of biopsies should be revised to include biopsies not only from distal duodenum but also from bulb to improve the diagnostic yield.

## Background

Celiac disease (gluten-sensitive enteropathy) is a chronic gastrointestinal disorder in which ingestion of gluten, a protein present in wheat, rye and barley, leads to damage of the small intestinal mucosa by an autoimmune mechanism in genetically susceptible individuals. Although highly sensitive serological tests are available for screening, small intestinal biopsies are essential to confirm the diagnosis of celiac disease [1].

The patchy nature of villous lesion in celiac disease is increasingly being recognized. The current guidelines (American Gastroenterological Association Technical Review 2006, North American Society for Paediatric Gastroenterology, Hepatology and Nutrition 2005) recommend that mucosal biopsies be taken from the second or more distal part of the duodenum for histological examination [2,3]. A minimum of four biopsy specimens is recommended. Multiple biopsies are needed to limit problems with orientation of the specimens and artefact during processing and staining.

Although the role of duodenal bulb biopsies in confirming celiac disease has been assessed in a few prospective research studies, no information is available on the outcome of applying this biopsy strategy in everyday, routine clinical practice where several different pathologists may be involved in reporting histological findings. The aim of the study was to investigate the usefulness of duodenal bulb mucosal biopsies in confirming the diagnosis of celiac disease in clinical practice.

## Methods

A previous report by Bonamico et al [4] showing the usefulness of duodenal bulb biopsies in the diagnosis of celiac disease had prompted the author (M.R.) to revise clinical practice by obtaining two additional biopsies from the duodenal bulb in cases of suspected celiac disease.

All patients with a positive tissue-transglutaminase (TTG) antibody test requiring biopsy-confirmation of celiac disease over a two-year period were retrospectively reviewed. The patients were all on a normal (gluten-containing) diet at the time of biopsy. Each patient had two endoscopic biopsies taken from the duodenal bulb and four biopsies from the second (or distal) part of the duodenum. Two biopsies were also taken from the gastric antrum for any *Helicobacter pylori *infection. The biopsy specimens were fixed in 10% formalin at the time of the endoscopy. All endoscopies were performed by a single gastroenterologist who was aware that the patient had a positive TTG test.

In the Pathology laboratory, the specimens had been routinely oriented and embedded in paraffin wax with standard sections at three levels. The duodenal histology was studied with haematoxylin and eosin stains. The intraepithelial lymphocytes (IELs) were counted using CD3 staining. The histology was reported using the modified Marsh criteria [5]. Marsh classification of the histologic changes of celiac disease include Type 0 or pre-infiltrative stage (normal), Type 1 or infiltrative lesion (increased intraepithelial lymphocytes i.e. >30 lymphocytes per 100 enterocytes), Type 2 or hyperplastic lesion (Type 1+ hyperplastic crypts), Type 3 or destructive lesion (Type 2 + variable degree of villous atrophy) and Type 4 or hypoplastic lesion (total villous atrophy with crypt hypoplasia). Type 3 has been modified to include Type 3a (partial villous atrophy), Type 3b (subtotal villous atrophy) and Type 3c (total villous atrophy). As is the routine in our institution, the pathologists studying the biopsies had been provided with a standard laboratory requisition with details of the tissue specimens and clinical information that the patient had a positive serological test and was being investigated for celiac disease.

The study was approved by the Research Ethics Board of the IWK Health Centre, Dalhousie University.

## Results

The review included thirty-five patients (14 males, 21 females). The mean age was 8.1 (± 4.7) years, range 1.3 to 18.7 years. The most common presenting symptoms were abdominal pain (40%) and diarrhoea (14.2%). In 20%, a screening serological test was done because the patient belonged to a high-risk population including having a positive family history, type-1 diabetes or Down syndrome. Most of these patients also had some mild symptoms.

Of the 35 patents, 31 (88.6%) had abnormal distal duodenal biopsies (Group I) and 4 (11.4%) had normal distal duodenal but abnormal bulb biopsies (Group II). The clinical, endoscopic and histological features of all patients (n = 35) are shown in Table 1.

**Table 1:** Clinical, endoscopic and histological features of all patients (n = 35).

No.	Age	Sex	Other Diagnoses	Duodenal Bulb		Distal Duodenum	
	(yrs)			Gross Appearance	Histology (Marsh type)	Gross Appearance	Histology (Marsh type)
**Group I**							

1)	12.5	M		friability	3b	mosaic, scalloping	3b
2)	3.7	M	Type 1 diabetes	normal	1	normal	3a
3)	1.8	M		normal	3c	normal	3c
4)	10.7	F	Type 1 diabetes	normal	1	normal	1
5)	11.9	M		normal	3a	mosaic, scalloping	3a
6)	7	F		edema	3b	mosaic, scalloping	3b
7)	2.2	M		normal	3c	mosaic, scalloping	3b
8)	13.8	M	Type 1 diabetes	normal	3a	mild scalloping	3a
9)	5.4	F		erythema	3a	normal	3a
10)	6.8	F		normal	3a	mild scalloping	3a
11)	2.5	F		nodularity	3b	scalloping	3b
12)	1.3	M		normal	3b	mosaic, scalloping	3b
13)	1.6	F		normal	3b	mosaic	3b
14)	10.3	M	IgA nephropathy	nodularity	0	scalloping	3b
15)	4.5	M		normal	3b	nodularity, scalloping	3b
16)	11.5	F	Type 1 diabetes	normal	3b	mosaic	3b
17)	7.3	F		normal	2	erythema, scalloping	2
18)	7.2	F	Down syndrome	nodularity	0	mosaic, scalloping	3a
19)	18.8	M	Type 1 diabetes	nodularity	3a	mosaic, scalloping	3a
20)	12.3	F		nodularity	3c	mosaic, nodularity	3c
21)	5.6	M		normal	3a	mosaic, nodularity	3a
22)	13.2	M		nodularity, mosaic	3c	normal	3b
23)	7.1	F	Type 1 diabetes	normal	3c	mosaic	3c
24)	2.6	F		normal	3a	scalloping	3a
25)	1.8	F		normal	3c	mosaic, scalloping	3c
26)	5.4	M		mosaic	3c	mosaic	3a
27)	14.2	F		mosaic	3c	scalloping	3b
28)	11.1	F		nodularity, mosaic	3c	scalloping	3a
29)	9	F		normal	3a	mosaic, scalloping	3a
30)	17.7	F	Type 1 diabetes	normal	3a	mosaic	3a
31)	10.1	F		normal	3b	normal	3b

**Group II**							

1)	10.8	F	Type 1 diabetes	nodularity	3a	normal	0
2)	9.2	M		mild erythema	2	normal	0
3)	9.4	F		mosaic	3c	normal	0
4)	4.6	F		normal	3c	normal	0

All patients had a positive TTG antibody.

In the thirty-one patients from Group I, one patient had Marsh type 1, one had Marsh type 2 and twenty-nine had Marsh type 3 lesion. All but two patients with abnormal distal duodenal biopsies also had abnormal bulb biopsies. Both these patients had Marsh type 0 lesion in the bulb and Marsh type 3 in the distal duodenum.

The distal duodenum showed gross abnormalities in 25 (81%) of cases in Group I. These are listed in Table 1 and included isolated scalloping of mucosal folds (n = 7), mosaic pattern (n = 5), and a combination of scalloping and mosaic appearance (n = 9). The remaining cases showed various combinations of nodularity, erythema and ulcerations, along with the above changes. Gross abnormalities of the duodenal bulb were present in 12 (38.7%) patients in this Group. These included nodularity (n = 5), mosaic appearance (n = 2), and a combination of both changes (n = 2). Mucosal friability, edema and erythema were present in one patient each. None of the patients in the group had *Helicobacter pylori *infection. There was no concern raised in the Pathology reports regarding a lack of sufficient biopsy tissue in any patient.

Group II consisted of four patients who had normal distal duodenal biopsies (Marsh type 0) but abnormal bulb biopsies. Of these four patients, one had Marsh type 2 and the other three had Marsh type 3 lesion. The clinical and pathological characteristics of these patients are shown in Table 1. Grossly, the duodenal bulb was completely normal in one patient; the other three had mild changes with patchy erythema, nodularity and mosaic appearance with friability respectively. The distal duodenum was grossly normal in all. None of the patients in the group had *Helicobacter pylori *infection.

Marsh type 1 (infiltrative changes) is non-specific and not diagnostic for celiac disease. The patient # 4 in Group 1 had only Marsh type 1 both in the bulb and distal duodenum. However, this patient had type-1 diabetes (a high risk group for celiac disease) and abdominal pain with a positive TTG antibody. Given all these factors, it was felt that the patient most likely has celiac disease. The evidence that Marsh type 2 (hyperplastic changes) is a distinctive feature of celiac disease is not very clear. The presence of Marsh type 2 changes on intestinal biopsy is suggestive of celiac disease and the diagnosis is strengthened by the presence of positive serological tests (3). The patient in Group I with Marsh type 2 lesion had abdominal pain, slow growth, anemia and a positive TTG antibody. The patient in Group II with Marsh type 2 lesion had poor growth and a positive TTG antibody. It was felt that there was enough clinical evidence of these being cases of celiac disease and treatment with gluten-free diet with observation of response to therapy is warranted.

**Figure 1:**
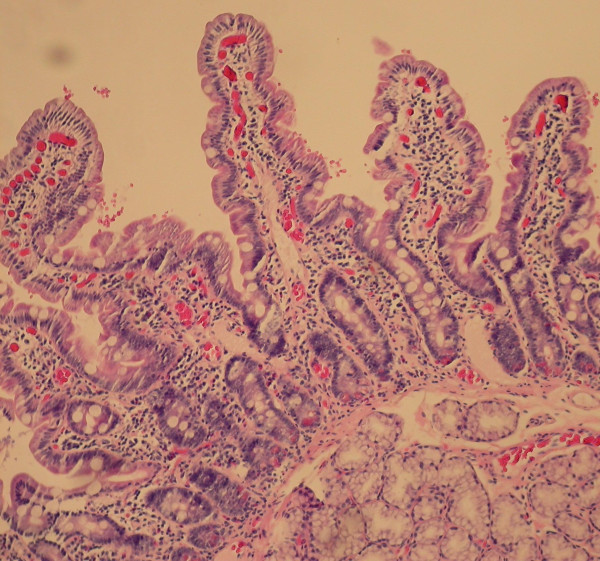
**Distal duodenal biopsy of patient #1 in Group II with celiac disease (hematoxylin & eosin stain)**.

**Figure 2:**
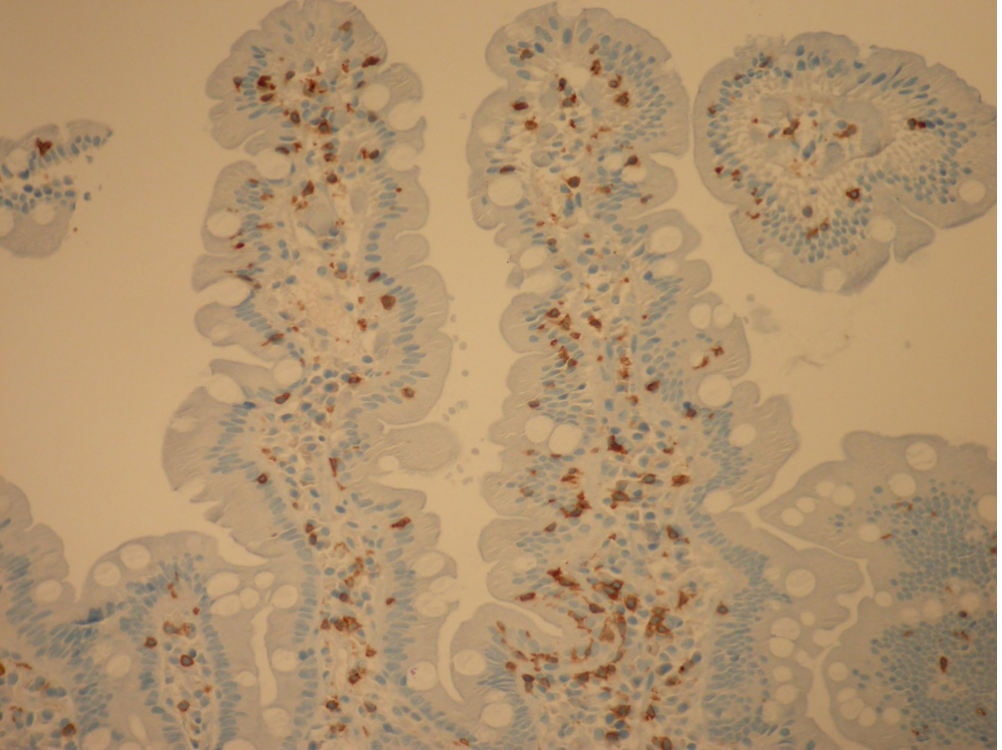
**Distal duodenal biopsy of the same patient in Group II with celiac disease showing intraepithelial lymphocytes (CD3 stain)**.

**Figure 3:**
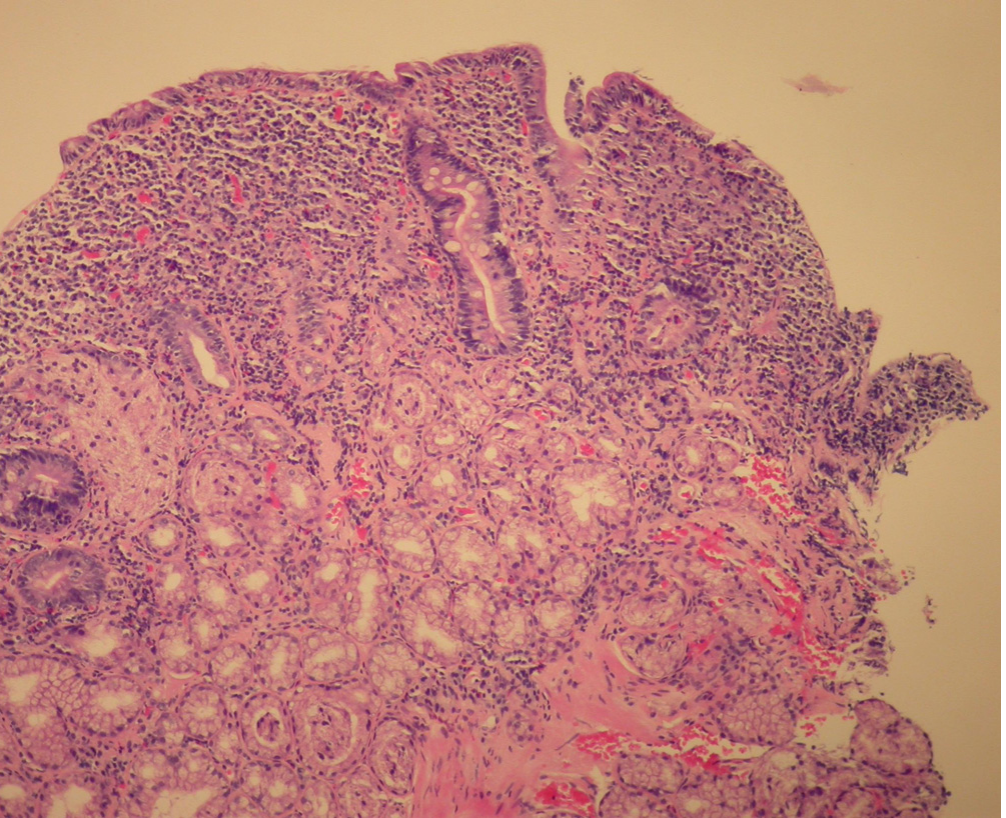
**Duodenal bulb biopsy of patient #1 in Group II with celiac disease (hematoxylin & eosin stain)**.

**Figure 4:**
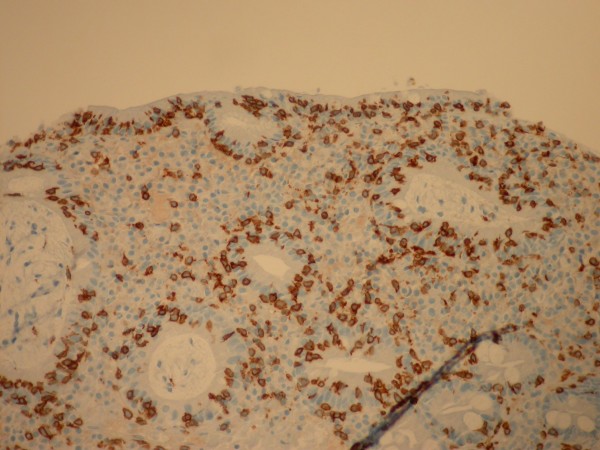
**Duodenal bulb biopsy of the same patient in Group II with celiac disease showing intraepithelial lymphocytes (CD3 stain)**.

The histology of the distal duodenum and duodenal bulb from patient number 1 of Group II is shown in Figures 1, 2 and 3, 4 respectively. The routine histology and the IEL count from the biopsies taken from distal duodenum were normal (Figures 1 and 2). Figure 3 shows the histology of the duodenal bulb mucosa of the same patient. There is total villous atrophy (Marsh type 3) with a marked increase in the IELs. This is further confirmed by the CD3 staining showing almost one IEL per enterocyte (Figure 4).

The histological confirmation of celiac disease would not have been possible in the four cases in Group II if only distal duodenal biopsies had been obtained. A combination of biopsies from the bulb and the distal duodenum identified celiac disease in all 35 patients suspected of the disorder.

## Discussion

The diagnosis of celiac disease is confirmed by the demonstration of inflammatory changes in the small intestinal villi. Since the duodenum and the proximal jejunum are exposed to the highest concentration of gluten, changes are more marked in the proximal small intestine than the distal. In the past, distal duodenal/upper jejunal biopsy was obtained using Crosby-Watson capsule. However, with fibreoptic endoscopy, the duodenum is much more easily accessible for mucosal biopsies. Multiple biopsies are recommended to help reduce the chances of insufficient tissue for histological assessment. Biopsies should be taken even if the duodenal mucosa grossly appears normal as histology may reveal disease in these cases [6].

Traditionally, for celiac disease, biopsies from the duodenal bulb have not been recommended on the assumption that the histology from this area may be difficult in interpret. The bulb contains more Brunner's glands and lymphoid tissue and can have gastric metaplasia compared to the distal duodenum [7]. The villi may also be shorter and broader in this area [8,9]. Duodenitis from other causes may also interfere with interpretation of villous atrophy in this region. The current guidelines by the professional gastroenterological organizations including American Gastroenterological Association (Technical Review 2006), North American Society for Paediatric Gastroenterology, Hepatology and Nutrition (Practice Guidelines 2005) and World Gastroenterology Organization (Practice Guidelines 2007) recommend four biopsies to be taken from the distal duodenum for histological examination in celiac disease [2,3,10].

Since the first classification of the spectrum of villous lesions in celiac disease by Marsh [11], there have been several modifications to this criterion. Counting of IELs can improve the diagnostic yield when the typical villous atrophy is not present or not detectable due to poor orientation or tangential cutting.

The patchy nature of the small intestinal lesion in celiac disease is increasingly being recognized both in children and adults [4,7,12-16]. The patchiness of lesion in various parts of the duodenum can be in terms of absence or presence of villous atrophy [4,12,13] or in the severity of atrophy [10]. Since the treatment of celiac disease requires a lifelong, strict adherence to gluten-free diet, making a definitive diagnosis of the disorder is of great importance.

In an earlier study, Bonamico et al [4] demonstrated the patchy nature of the lesion in celiac disease both in children who were newly diagnosed and those on a gluten challenge. In all 95 children at the time of diagnosis of celiac disease, the bulbar mucosa was involved showing varying degrees of type 3 villous atrophy. In four (4.2%) patients, the bulb was the only duodenal area involved with the other duodenal samples being normal. Prasad et al have reported similar findings of duodenal bulb involvement [13]. In 52 children with suspected celiac disease who underwent one bulb and one distal duodenal biopsy, all had Marsh type 3 lesions in at least one of the sites. The authors concluded that duodenal bulb biopsy was equally diagnostic of celiac disease. More recently, in a large Italian study of children with celiac disease the duodenal bulb was involved in all cases of and in some patients the lesion was only presented in the bulb with distal duodenum being normal [14]. Villous atrophy limited to duodenal bulb has also been described in adults with celiac disease [15,16].

Our study confirms this finding of distal duodenal sparing in a significant number of patients (11.4%) with celiac disease. However, in our study, of the 31 patients with abnormal distal duodenal biopsies, only 29 had abnormalities in the bulb while other 2 had normal bulb mucosa. This testifies further to the patchy nature of the villous lesion in celiac disease. It is also important to note that there could be bulb sparing in some patients, albeit few. This is in contrast to other reports where bulb was involved in all cases [12-14]. We conclude that biopsies taken from the bulb alone are not enough as they will miss some patients.

Recognizing the patchy nature of the disorder, Hopper et al further studied the number and location of biopsies required to make a definitive diagnosis of celiac disease [17]. Nine biopsies were taken; one from the bulb, four from proximal duodenum and four from distal duodenum. These were evaluated individually based on their ability to identify villous atrophy, and on their success when combined. It was determined that all of the optimal combinations of biopsy sites included a duodenal bulb biopsy.

Previous studies had demonstrated the usefulness of duodenal bulb biopsies in research settings with a single pathologist reporting while blinded. The present study is the first one to examine this practice in routine, everyday clinical care. A major difference between our and previous studies is having more than one pathologist involved in interpreting the biopsies taken from patients in our study. There were four pathologists reporting on different patients. All were experienced, academic paediatric pathologists. Also, none of the pathologists were blinded. In real life, Pathology Departments of health care institutions will have several pathologists involved in interpreting small intestinal biopsies for celiac disease. It is not known how often pathologists in academic or community hospitals do IEL counting routinely. Also, pathologists may use different modifications of Marsh criteria, although it is hoped that each pathologist follows one particular classification to keep consistency in interpretation of the biopsies. Moreover, the possibility that different pathologist interpret duodenal biopsies differently cannot be excluded. Similarly, one cannot discount the possibility of an inaccurate interpretation of the biopsies by a given pathologist. Interpretation of small intestinal biopsies for celiac disease requires experience and familiarity with the spectrum of the histological changes. Knowledge of the clinical history and the working diagnosis of celiac disease may also bias the pathologists in their interpretation of the findings. Insufficient tissue or poor orientation of the biopsy specimens can also affect interpretation. However, these phenomenon are likely to be present and persist in real-life clinical practice. It is important to point out that our study is a retrospective review of biopsy results as reported by the attending pathologist. The aid of another pathologist for a second opinion was not sought.

Based on the current and previous studies, we recommend that biopsies should be taken both from the bulbar and the distal duodenal mucosa, as these will complement each other in confirming the diagnosis of celiac disease. Accepting that villi in the bulb may be less tall, increased IELs in the presence of a positive serological test will help improve the likelihood of the diagnosis of celiac disease. We concur with the suggestion by Hopper et al that multiple biopsy strategy should incorporate a biopsy from the duodenal bulb [17]. Moreover, the bulb should be biopsied irrespective of its gross appearance. We speculate that some patients considered to have a "false-positive" serological test may, in fact, truly have celiac disease. The diagnosis could have been missed in these cases as the biopsies are taken routinely only from the distal duodenum and not from the bulb.

## Conclusion

In conclusion, this study confirms previous reports that villous atrophy can be patchy in pediatric patients with celiac disease with duodenal bulb mucosa being the only area showing histological changes in some cases. The current recommendations regarding the site of biopsies need to be revised as they may lead to a false-negative diagnosis with significant implications for the patient. The optimal strategy for detecting villous changes should include biopsies not only from the distal duodenum but also from the bulb to improve the diagnostic yield. Biopsies taken from both sites can confirm histological diagnosis in all cases of celiac disease in clinical practice.

## Competing interests

The authors declare that they have no competing interests.

## Authors' contributions

MR conceived of the study, designed it and drafted the manuscript. AM gathered the data. All authors participated in analyzing the data and read and approved the final manuscript.

## Pre-publication history

The pre-publication history for this paper can be accessed here:

http://www.biomedcentral.com/1471-230X/9/78/prepub
